# Potential selection for lipid kinase activity and spermatogenesis in Henan native pig breeds and growth shaping by introgression of European genes

**DOI:** 10.1186/s12711-023-00841-y

**Published:** 2023-09-18

**Authors:** Ruimin Qiao, Xinjian Li, Ole Madsen, Martien A. M. Groenen, Pan Xu, Kejun Wang, Xuelei Han, Gaiying Li, Xiuling Li, Kui Li

**Affiliations:** 1 College of Animal Science, Henan Agricultural University, Zhengzhou, 450046 China; 2 Jiangsu Agri-Animal Husbandry and Veterinary College, Taizhou, 225300 China; 3 Animal Breeding and Genomics Centre, Department of Animal Sciences, Wageningen University & Research, 6700 HB Wageningen, The Netherlands; 4 State Key Laboratory of Animal Nutrition and Key Laboratory of Animal Genetics, Breeding and Reproduction of Ministry of Agriculture and Rural Affairs of China, Institute of Animal Sciences, Chinese Academy of Agricultural Sciences, Beijing, 100081 China

## Abstract

**Background:**

China has one third of the worldwide indigenous pig breeds. The Henan province is one of the earliest pig domestication centers of China (about 8000 years ago). However, the precise genetic characteristics of the Henan local pig breeds are still obscure. To understand the origin and the effects of selection on these breeds, we performed various analyses on lineage composition, genetic structure, and detection of selection sweeps and introgression in three of these breeds (Queshan, Nanyang and Huainan) using genotyping data on 125 Queshan, 75 Nanyang, 16 Huainan pigs and 878 individuals from 43 Eurasian pig breeds.

**Results:**

We found no clear evidence of ancestral domestic pig DNA lineage in the Henan local breeds, which have an extremely complicated genetic background. Not only do they share genes with some northern Chinese pig breeds, such as Erhualian, Hetaodaer, and Laiwu, but they also have a high admixture of genes from foreign pig breeds (33–40%). Two striking selection sweeps in small regions of chromosomes 2 and 14 common to the Queshan and Nanyang breeds were identified. The most significant enrichment was for lipid kinase activity (GO:0043550) with the genes *FII*, *AMBRA1*, and *PIK3IP1*. Another interesting 636.35-kb region on chromosome 14 contained a cluster of spermatogenesis genes (*OSBP2*, *GAL3ST1*, *PLA2G3*, *LIMK2*, and *PATZ1*), a bisexual sterility gene *MORC2*, and a fat deposition gene *SELENOM*. Reproduction and growth genes *LRP4*,* FII*, and *ARHGAP1* were present in a 238.05-kb region on SSC2 under selection. We also identified five loci associated with body length (*P* = 0.004) on chromosomes 1 and 12 that were introgressed from foreign pig breeds into the Henan breeds. In addition, the Chinese indigenous pig breeds fell into four main types instead of the previously reported six, among which the Eastern type could be divided into two subgroups.

**Conclusions:**

Admixture of North China, East China and foreign pigs contributed to high genetic diversity of Henan local pigs. Ontology terms associated with lipid kinase activity and spermatogenesis and growth shaping by introgression of European genes in Henan pigs were identified through selective sweep analyses.

**Supplementary Information:**

The online version contains supplementary material available at 10.1186/s12711-023-00841-y.

## Background

Henan is one of the cradles of Chinese civilization and one of the earliest pig domestication sites in China. A large number of pig bones have been excavated from the early Neolithic site of Jiahu (9000–7500 years ago), located in the Wuyang County of the south-central Henan Province. Morphological, pathological, and population structure (age and sex ratio) analyses of 340 pig bone fragments, including the typical jaw bones, from the earliest period of this site (6500 BC), indicated that they originated from domestic pigs [1]. Analysis of food refuse remains showed that pork accounted for about 30% of the total meat consumed during this period, and reached 60 to ~ 70% in the middle and 80 to ~ 90% in the late period of the Peiligang and Yangshao cultures (5000 to 3000 BC). The Jiahu site is the earliest known pig domestication site in North China and is thousands of miles away from the site of Kuahuqiao (8200–7000 years ago), which is located in Xiaoshan [2] of the Zhejiang Province, the earliest pig domestication site in South China. Morphological differences between the bone remains of domestic pigs at these two sites coincide with the difference between wild boars living in the north and south of China. The wild boar from North China is large with a long snout, while that from South China is small with a short and broad snout [1]. The geographical locations of the two sites are shown in Additional file 1: Fig. S1.

In 1986, the local pig breeds in China were divided into six types according to their origin, production performance, morphological characteristics, geographical distribution, and socioeconomic conditions: North China, East China, Center China, South China, Southwest China, and Plateau China [3]. Briefly, North China covers mainly the vast area north of the Huai River and the Qinling Mountains, with convenient transportation, while the South China region is mainly located in the south of Nanling and in the Pearl River Basin. Center China covers mainly the vast area between the middle and lower reaches of the Yangtze River and Pearl River, with many hills. East China covers mainly the narrow transitional zone between the areas comprising the North Chinese and Center Chinese pigs, including the middle and lower reaches of the Yangtze River, coastal areas, and the coastal plain of western Taiwan Province. The Southwest China region covers most of the Sichuan Basin, the Yunnan-Guizhou Plateau, and the western areas of the Hunan and Hubei Provinces. The Plateau China region is mainly represented by the Qinghai-Tibet Plateau. Henan province harbors three traditional native pig breeds that are named Queshan, Nanyang, and Huainan, which were classified as North Chinese [3], with the main production areas being Zhumadian, Nanyang, and Xinyang. These three breeds have a black coat and lop ears, a medium size with a mature body weight of 90 to 120 kg that is comparable to that of other Chinese local pigs, and a litter size of 9 to 11 piglets. The Nanyang breed is characterized by a grey skin, harder ear roots, and a long and thick mane that is 9 to 15 cm long, while the Queshan and Huainan breeds have a stubby mane. The Henan breeds are less susceptible to diseases than commercial pig breeds, they can survive under poor management and crude feed [4–7], and similar to most Chinese local pigs, their meat is palatable.

From the early mid-nineteenth century, foreign pig breeds began to be introduced in China to improve the body shape of Chinese native pigs and from the mid-twentieth century, the People's Republic of China imported several commercial pig breeds such as Landrace, Yorkshire, Hampshire, Berkshire, Pietrain, and Duroc, and established foreign-trade pig farms, three of which were built in Nanyang and Zhumadian in Henan province. This province also introduced several commercial pig breeds during that period, such as Berkshire, Hampshire, Landrace, Yorkshire, and Duroc (see Additional file 2: Table S1) [8–11]. Thus, we hypothesized that the genome of the Henan pig breeds might have been influenced by long-term natural and artificial selection or recent interbreeding with foreign pigs.

Our previous population genetics study in which 41,763 single nucleotide polymorphisms (SNPs) were used to genotype nine Nanyang, nine Huainan, and 10 Queshan pigs from Henan province, and three Duroc, two Landrace, and three Yorkshire pigs, showed admixture of the Nanyang pigs with foreign pigs [12]. However, the comprehensive and accurate genetic characteristics of the present core groups of Henan pig breeds remain unclear. Thus, for the first time, we performed various analyses to study the phylogenetic relationships, ancestral lineage, historical admixture, genetic diversity, signatures of selection, and an association analysis of the selected signals in the core groups of three Henan local pig breeds, compared with 43 Asian and European-American pig breeds (see Additional file 3: Table S2).

## Methods

### Sample collection and genotyping

Ear and tail tissue samples from three Henan local pig breeds, Queshan (n = 130), Nanyang (n = 75), and Huainan (n = 18) were collected and conserved in 75% ethanol at − 40 ℃. Genomic DNA was extracted following the standard phenol–chloroform extraction procedure and genotyped with the GeneSeek Genomic Profiler Porcine HD BeadChip (68,516 SNPs) (Neogen Corporation, USA). The PorcineSNP60 BeadChip array v1 or v2 (Illumina, San Diego, CA, USA) SNP genotypes of 878 additional individuals from 43 Asian and European-American pig breeds (see Additional file 3: Table S2) were downloaded from previous studies [13–15]. In total, 42,464 autosomal SNPs in common between these two platforms were considered for further analysis. SNPs with a minor allele frequency (MAF) lower than 0.01, those with more than one position in the latest 11.1 version of the pig genome or a call rate < 90% were filtered out using the PLINK v1.9 software, which was also used to merge and prune the genotyping data [16]. After quality control, the genotypes of 34,932 autosomal SNPs for 1094 pigs remained for further analyses, including 125 Queshan, 75 Nanyang, 16 Huainan pigs, and 878 pigs from 43 Eurasian breeds from the public database.

### Analysis of the genetic relationships among Henan and Eurasian pigs

The PLINK software was used to calculate the matrices of identity-by-state (IBS) distances and genetic differentiation (fixation index, *F*_ST_) [17] between each pair of pig breeds in order to estimate phylogenic relationships between breeds. The IBS matrix was applied to build neighbor-joining trees for all individuals using the PHYLIP v3.69 software (https://evolution.genetics.washington.edu/phylip.html), and Figtree v1.4.2 (http://tree.bio.ed.ac.uk/software/figure/) was used to view the trees. Based on the pairwise *F*_ST_ matrix, we reconstructed the phylogenetic relationships and network using the neighbor-net algorithm of SplitsTree v4.14.6 [18]. Principal component analysis (PCA) was performed with the GCTA (http://cnsgenomics.com/software/gcta/#Overview) [19] and R software.

### Inference of ancestral lineage and historical admixture

To investigate the genetic background of the 46 analyzed pig breeds, and especially that of the core groups of the Henan native pigs, 10 pigs from each breed were randomly selected. The genotypes of 26,169 qualified SNPs of these individuals with linkage disequilibrium (LD) (r^2^) values lower than 0.5 were filtered to carry out population structure analysis using the ADMIXTURE v1.3.0 software [20] with K values ranging from 2 to 46. The optimal K number was determined by cross-validation error. The TreeMix [21] software was used to construct a maximum likelihood tree to infer population splitting and mixing. Three-population statistics (*f*3) were calculated using the *threepop* program that is included in TreeMix to determine if a population was a mixture of two other populations. Duroc was defined as an outgroup with a Z-score lower than − 2 being significant. The ROLLOFF software, which is part of the ADMIXTOOLS package [22, 23], was used to date the admixture events based on the rate of exponential decay of admixture-induced LD and to date the introgression events based on the results of the *f*3 statistics.

### Genetic diversity analysis

To investigate the genetic diversity within the 46 pig breeds, the expected heterozygosity (He), observed heterozygosity (Ho), and LD decay were calculated with the PLINK v1.9 software, using default settings [24]. Effective population size (Ne) was estimated from LD data using SNeP v1.11 [25] by applying sample size correction for phased genotypes and Sved and Feldman's recombination rate modifier [26]. A measure of the inbreeding level of each population was obtained by the average individual inbreeding coefficient (F) and the genomic inbreeding (F_ROH_), which was calculated as the proportion of the genome in runs of homozygosity (ROH). The ROH of each individual were detected by PLINK v 1.9 using a 1000-kb sliding window containing at least 15 SNPs [24]. Non-heterozygous SNPs and one missing call per window were allowed to avoid false negatives.

### Detection of signatures of selection

Selection sweep analysis was performed in 125 Queshan and 75 Nanyang pigs based on both haplotype and genetic differentiation (*F*_ST_) strategies using 53,685 qualified SNPs. First, we calculated the frequencies of all SNPs in ROH segments that were identified in the Queshan and Nanyang pigs; a Manhattan plot of the frequencies against the chromosomal position of the corresponding SNPs was built. The top 1% SNPs in ROH (empirical distribution) were defined as significant loci that were putatively under selection [27]. Then, the integrated haplotype homozygosity pooled test (iHH12) [28] was implemented to detect signatures of selection in the Queshan and Nanyang pigs using the selscan v1.2.0a program, using default parameters [29]. The haplotypes were phased by Beagle v4.0 [30]. The iHH12 scores were normalized by the *norm* software using default parameters [29] and presented as a Manhattan plot. The top 1% SNPs (empirical distribution) were defined as potentially selected loci. The metascape (https://metascape.org/) [31] database was used to achieve a better understanding of the biological functions of the regions under selection that overlapped between the ROH and iHH12 analyses.

In addition, *F*_ST_ values for the Queshan and Nanyang pigs were compared with those of eight representative Chinese pig breeds based on the results of the ADMIXTURE analysis to determine if there were any unique signatures of selection in the Henan breeds. SNPs that were in the top 1% empirical distribution for *F*_ST_ values [27] were assumed to be candidate loci.

### Genetic association analysis

The allele frequencies of the SNPs that were shared between the ROH and iHH12 analyses, and that were in common with the candidate SNPs from the *F*_ST_ analysis of signatures of selection in the Queshan and Nanyang pigs, were calculated separately for the three Henan breeds, the eight representative Chinese breeds, and six foreign breeds in order to uncover the origin of these likely loci under selection. To investigate the effects of these loci, they were used to genotype 365 nucleus Sujiang individuals for which body size traits at the age of 180 ± 5 days were available. Briefly, body length was defined as the distance from the middle of the ears to the root of the tail and was measured by a meter ruler when pigs stood naturally. The Sujiang pigs were raised in a provincial breeding farm under the same feeding conditions. We used a mixed linear model with body weight and batch as fixed effects to test the associations of these common SNPs with body size traits with the R software.

## Results

### Genomic signatures of admixture from foreign pigs to Henan native pigs

**Fig. 1:**
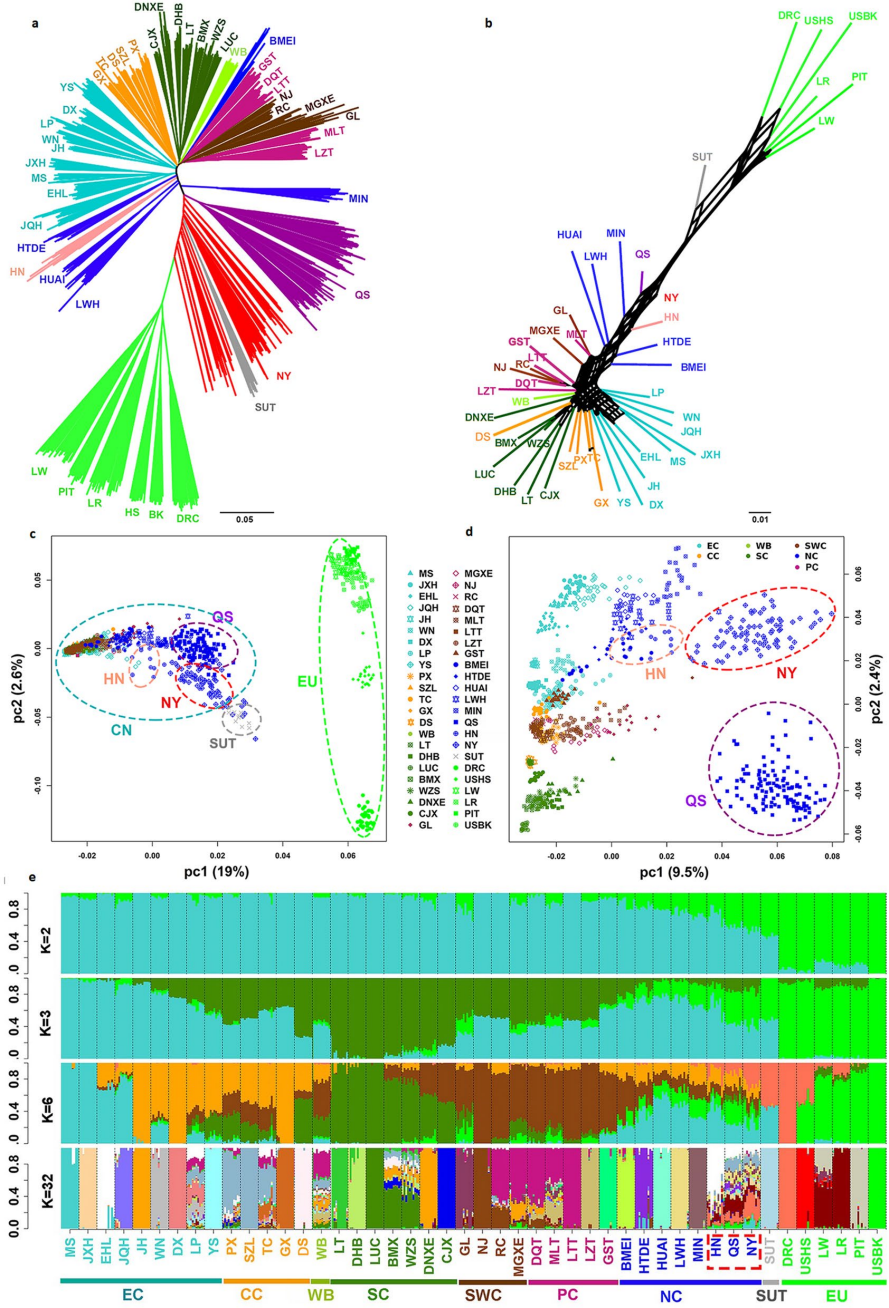
Phylogenetic relationship and population structure of the three Henan breeds compared with 43 Eurasian breeds. **a** IBS-based neighbor joining tree; **b** *F*_ST_ based neighbor-net tree. The scale bar represents a 0.01 base substitution per site; **c** Principal component of the 46 Eurasian breeds; **d** Principal component of 39 Chinese indigenous breeds; and **e** Ancestral lineage compositions of the 46 Eurasian breeds revealed by ADMIXTURE analysis. K values on the Y-axis are the number of hypothetical ancestral populations. Full name of each breed is shown in Additional file 2: Table S1. NC: North China, EC: East China, CC: Center China, SC: South China, SWC: Southwest China, PC: Plateau China [3]

To get a phylogenetic overview of the Henan pig breeds, we constructed a neighbor joining tree (Fig. 1a) and a neighbor-net splits network (Fig. 1b) based on 1094 individuals from 40 Chinese pig breeds and six foreign pig breeds. We found that the Chinese local breeds and the foreign breeds were situated at each end of the phylogenetic tree, while the Sutai breed, which is a Chinese synthetic breed that was derived from Duroc boars and Taihu sows for more than 20 generations, is located in the middle of the tree. The length of the branches for the foreign pig breeds was more uniform and longer (Fig. 1a and b) than that of the Chinese pig breeds. Figure 1a shows that individuals from the same breed clustered together, except for the Nanyang individuals, and that the six types of Chinese local pig breeds clustered in separate groups in the neighbor joining tree, except for the breeds from North China and Plateau China. The distributions of the pig breeds from North China were more dispersed than those of the five other breed types, especially the Bamei and Nanyang breeds. The branch corresponding to the Bamei breed, which lives at high altitude, was near to that of the breeds from Plateau China, while the branch of the Nanyang breed was close to that of the foreign pig breeds. Some of the Nanyang pigs were even situated between the Sutai and foreign pig breeds. In addition, the cluster of Queshan pigs had two sub branches. East Chinese pigs in peacock blue were also divided into two main subclusters: (1) one sub branch was formed by the four all-black breeds: Jiaxing black, Meishan, Erhualian, and Jiangquhai, which are geographically next to the North Chinese pigs; and (2) a second sub-branch formed by three spotted breeds, Wannan, Leping, and Dongxiang, one two-end-black breed, Jinhua, and one all-black breed, Yushan, which are geographically close to the breeds from Center China pigs (see Additional file 1: Fig. S1). The breeds from Center China include most of the Huazhong two-end-black pig breeds and some piebald pig breeds. The breeds from Center China and from South China showed good aggregation. The breeds from Plateau China were separated into two clusters by the four breeds from Southwest China.

The neighbor-net split network (Fig. 1b) shows the dispersion of the breeds from North China, especially the Queshan and Nanyang breeds from the Henan province, the deviation of the Bamei breed from the breeds from North China, and the inseparability between the breeds from Plateau China and Southwest China. Although the two-end-black breed Dongshan clustered with the breeds from Center China, in the neighbor-net split network it clustered with its geographical neighbor breeds from South China (Fig. 1b). Several parallel splits are observed in the networks of both the foreign and Chinese pig breeds, especially the breeds from East China, which indicates that these breeds have responded differently to genetic selection. The breeds from South China seem to be placed at the root position of the Chinese native pig breeds, followed by the breeds from Center China.

In order to investigate the genetic structure of the 46 pig populations analyzed in our study, we carried out PCA and admixture analyses. To specify the stratification of the Chinese local pig populations, we conducted a PCA on 39 Chinese pig breeds, excluding the Sutai breed. The results of the PCA and phylogenetic analyses were similar, revealing a significant separation between the Chinese and the foreign pig breeds, the middle position of the Sutai breed, the dispersion of the breeds from North China, especially the Queshan and Nanyang local breeds from the Henan province and the Bamei breed, and the existence of two subgroups of breeds from East China. A separation between the foreign pig breeds is also observed (Fig. 1c and d).

Next, we randomly selected 10 individuals from each of the 46 breeds to reduce sample bias and perform the admixture analysis (Fig. 1e). Chinese and foreign pig populations were separated at K = 2, with some of them sharing a small number of genes. The North Chinese type had a much higher proportion of foreign genes than the other five types, especially the Nanyang and Queshan pigs of the Henan province. Admixture analysis of 10 individuals at a time revealed that the Huainan, Queshan, and Nanyang pigs contained 33, 41, and 40% of foreign genes. At K = 3, a new ancestry for the Chinese pigs was detected, represented by the breeds from South China. The exotic lineage in foreign pigs had the same ancestral origin as the South Chinese pig breeds. The Henan local pigs shared nearly the same lineage with the East Chinese pigs, and also shared lineage with some foreign and South Chinese pigs.

At K = 6, the commercial pig breeds were divided into Duroc and Berkshire origins, which are both present in the Henan pig breeds. Two new local Chinese pig ancestral origins appeared, in earth yellow and dark brown. These results confirm those of the phylogenetic and PCA analyses. For example, the Sutai breed, with a 50% Duroc lineage and 50% East Chinese lineage, and the breeds from East China were divided into two subgroups: one formed by the Meishan, Jiaxing black, Erhualian, and Jiangquhai breeds, and one by the Jinhua, Wannan, Dongxiang, Leping, and Yushan breeds. In addition, the Dongshan breed from Center China shared more ancestry with the breeds from South China, while the Bamei breed from North China shared more ancestry with the breeds from Plateau China and Southwest China. Thus, the lineage composition of the Henan pig breeds is complex since they include not only all four Chinese local pig lineages origin (East China, Center China, Southwest China, Plateau China, and South China), but also all two kinds of foreign pig lineage origins. The Nanyang breed had the highest level of Duroc lineage ancestry.

At K = 32, which was determined by cross-validation error to be optimal, six foreign pig breeds contained five ancestral lineages, three of which shared lineage with the Large White breed colored in brick red. Some Chinese native pig breeds had many kinds of lineage origins, such as the wild boar, the Leping, Bamaxiang, Wuzhishan, Mingguangxiaoer, Diqing Tibetan, Milin Tibetan breeds, and the Queshan and Nanyang breeds from the Henan province. The Huainan breed from the Henan province had almost the same ancestry as the Erhualian breed.

### Estimated origin and timing of introgression events in the Henan indigenous breeds

**Fig. 2:**
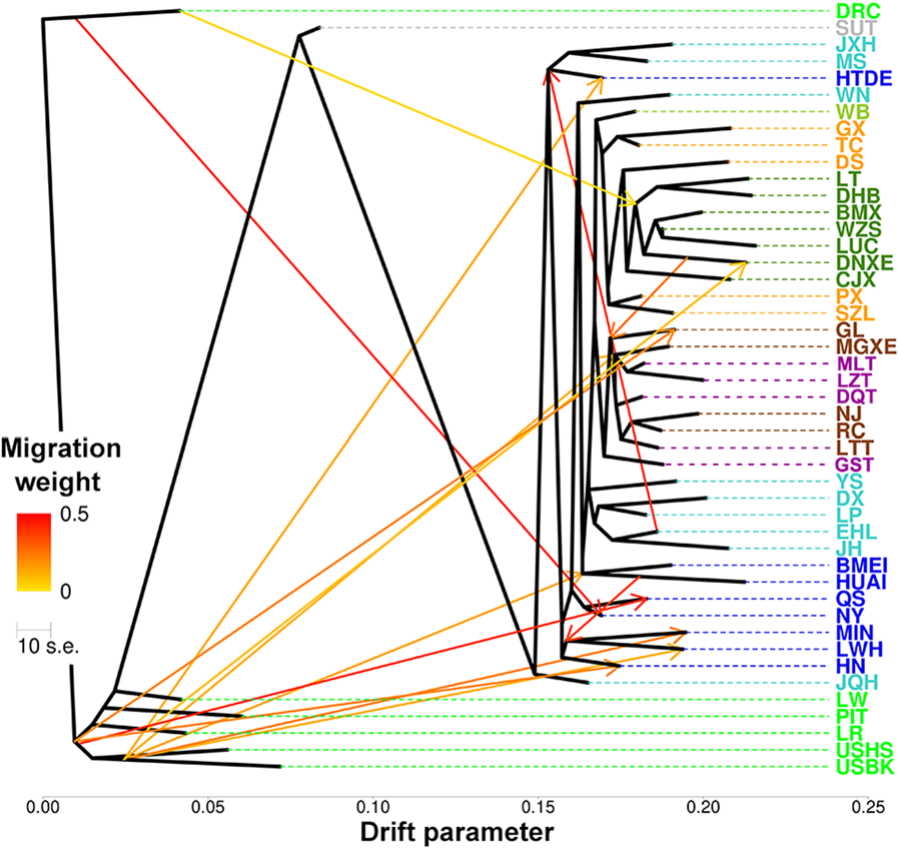
Historical migration events between 46 Eurasian breeds revealed by TreeMix analysis. The full name of each breed is in Additional file 2: Table S1

To detect gene migration events in the Henan pig breeds, we used Duroc pigs as the outgroup. When setting 14 migration events, the maximum likelihood tree explained 99.99% of the variation in 46 Eurasia-American pig breeds (Fig. 2). Eleven of the 14 migrations were from foreign pig breeds to Chinese pig breeds and the remaining three were between Chinese pig breeds, including migrations from Erhualian to Hetaodaere, Huai to Min, and Laiwu, and Diananxiaoer to Huanling. Migrations from foreign pigs were detected for all three Henan pig breeds (Huainan, Queshan, and Nanyang).

To evaluate the extent of gene mixture in the genome of the Henan pig breeds, we chose the Erhualian, Neijiang, and Luchuan pig breeds and the Duroc and Berkshire breeds for the *f*3 analysis. Twenty significant combinations of pig breeds were obtained (see Additional file 4: Table S3). The three breeds from the Henan province (Huainan, Queshan and Nanyang) all had admixture from Duroc and Berkshire. To further estimate the time of the admixture events, we calculated the average timing of the 20 significant admixture combinations using the ROLLOFF program. The admixture event with Berkshire was detected to have occurred earlier than that with Duroc (Table 1). In view of a generation interval of about 4 years in Chinese local pigs, the times of the admixture events of Berkshire and Duroc to the Huainan breed (51–63 and 40–50 years ago, respectively) and to the Queshan breed (51 to 64 and 33 to 41 years ago, respectively) were close and about four generations earlier than those to Nanyang (30 to 38 and 17 to 21 years ago, respectively).

**Table 1:** Origin and timing of the introgression events in the Henan indigenous pigs

Breed	Origin	Admixture generation (mean)	Timing (years ago)
Huainan	Berkshire	12.63	50.52–63.15
	Duroc	9.89	39.56–49.45
Queshan	Berkshire	12.81	51.24–64.05
	Duroc	8.28	33.12–41.40
Nanyang	Berkshire	7.50	30.00–37.50
	Duroc	4.24	16.96–21.20

### Effect of the admixture of European DNA on genetic diversity of the Henan indigenous pig breeds

**Fig. 3:**
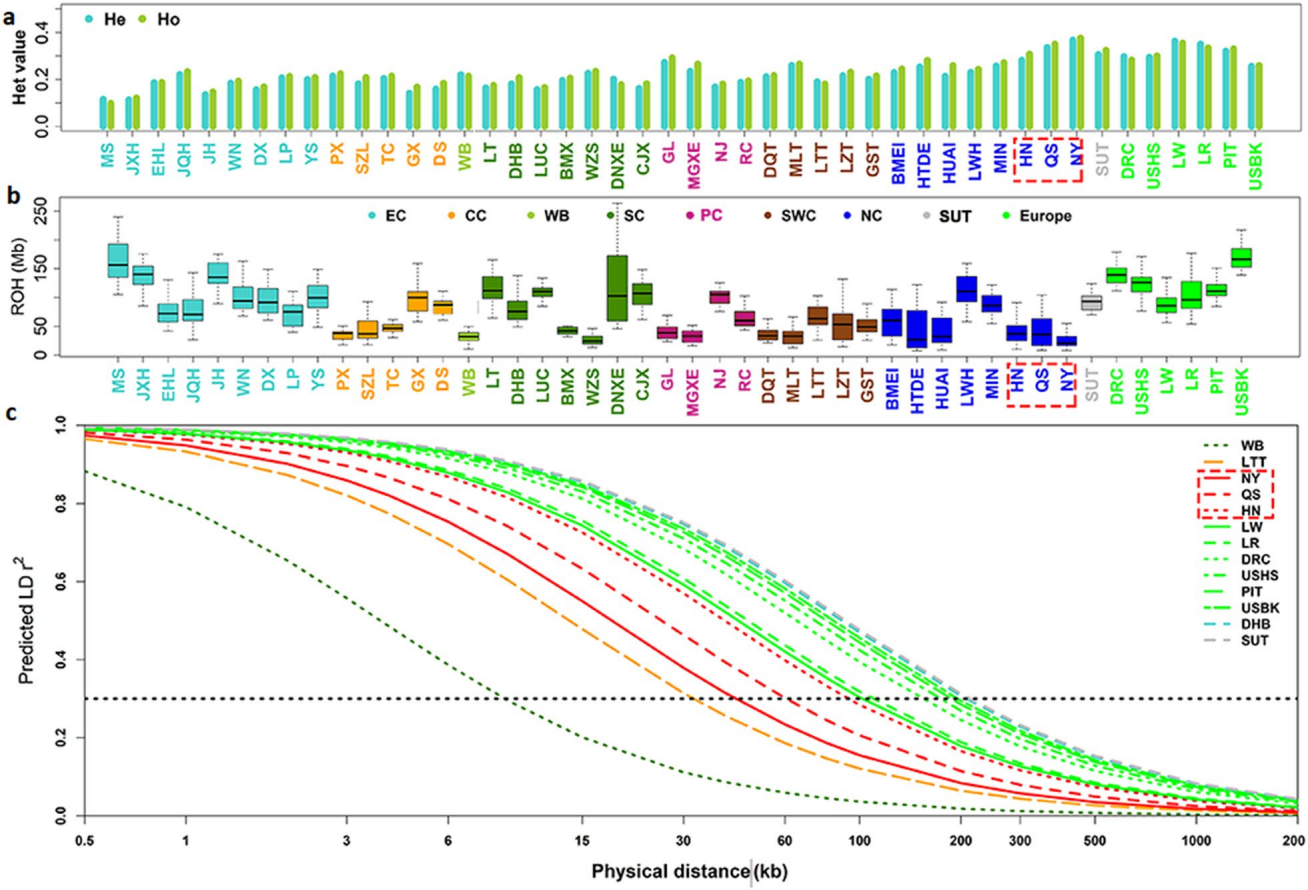
Genetic diversity of 46 Eurasian pig breeds used in this study. **a** Expected heterozygosity (He), observed heterozygosity (Ho); **b** runs of homozygosity (ROH); and **c** linkage disequilibrium (LD) decay patterns of 13 representative pig breeds. Henan indigenous pig breeds are indicated by a red box. The full name of each breed is given in Additional file 2: Table S1

To analyze the effects of admixture with foreign pigs on the genetic diversity of the local breeds from the Henan province, we compared estimates of Ho, He, Ne, No (observed population size), F, ROH, F_ROH_, and LD decay for the 1094 individuals included in our study. Based on the results in Fig. 3a and Additional file 3: Table S2, the average He and Ho were highest (0.271 and 0.292) and lowest (0.169 and 0.174) for the North China and East China breed types, respectively. The estimates of He and Ho for the three Henan breeds, i.e. (0.282 and 0.308 for Huainan, 0.336 and 0.35 for Queshan and 0.367 and 0.377 for Nanyang, were the highest among the breeds from North China and similar to those of the Sutai (0.307 and 0.325) and foreign pig breeds. The lowest average Ne/No (2.65) was found for the breeds from North China (see Additional file 3: Table S2), especially for the Nanyang (1.2) and Queshan (0.6) breeds. Wild boars had the highest Ne/No (7.90).

The average inbreeding coefficient F was lowest for the Nanyang (− 0.029) and Queshan (0.038) breeds, resulting in a lower average F value for breeds from North China than for the other five Chinese native breed types. Among the six types of Chinese native pigs, those from North China had the lowest F_ROH_, with the Nanyang breed having the lowest value (0.01). Compared to the average F_ROH_ of the Chinese native pig breeds (0.030) (except wild boar) (Fig. 3b and see Additional file 3: Table S2), the Huainan and Queshan breeds also had very low F_ROH_ (0.017 and 0.018, respectively).

At an average LD coefficient (r^2^) of 0.3, the genetic distance between molecular markers was shortest for wild boars (8.83 kb), followed by Litang Tibetan (32.13 kb) (Fig. 3c, and see Additional file 3: Table S2). The lengths of LD decay were shorter for the breed types from Southwest China (52.76 kb) and Plateau China (76.16 kb) than for the other four types of Chinese pig breeds (from 104.70 kb to 133.66 kb). The Nanyang (42.82 kb), Queshan (60.49 kb) and Huainan (92.81 kb) breeds had a relatively shorter LD decay than the other Chinese breeds. The Sutai breed had the longest average distance between 10 neighboring SNPs (212.28 kb), followed by the Dahuabai (207.22 kb) and the six foreign pig breeds.

### Genomic signatures of selection and their contribution to breed characteristics of the Henan indigenous pig breeds

Domestic pigs from the Henan province have experienced not only strong artificial and natural selection for thousands of years, but also admixture with foreign pigs. Thus, we further searched for selection traces in the Queshan and Nanyang breeds using ROH and iHH12 strategies based on haplotypes. For the Queshan breed, the ROH and iHH12 analyses detected 551 and 506 significant loci (top 1%), respectively, of which 153 were in common and located on seven chromosomes, covering 92 annotated genes. Similarly, for the Nanyang breed, the ROH and iHH12 analyses detected 534 and 524 significant loci, respectively, of which 130 were in common and located on nine chromosomes, covering 73 annotated genes. The Venn diagram of these loci is shown in Additional file 5: Fig. S2A.

Gene ontology (GO) and Kyoto Encyclopedia of Genes and Genomes (KEGG) enrichment analyses of the above genes were performed in Metascape and are presented in Additional file 6: Fig. S3. The 92 genes identified in the Queshan breed were mainly enriched in the categories: dendrite (GO:0030425), regulation of lipid kinase activity (GO:0043550), hsa04140, autophagy, acute-phase response (GO:0006953), and spermatogenesis (GO:0007283) (see Additional file 6: Fig. S3A). The 73 genes identified in the Nanyang breed were mainly enriched in the categories: regulation of lipid kinase activity (GO:0043550), regulation of glial cell differentiation (GO:0045685), cellular response to nutrient levels (GO:0031669), and gamete generation (GO:0007276) (see Additional file 6: Fig. S3B).

**Fig. 4:**
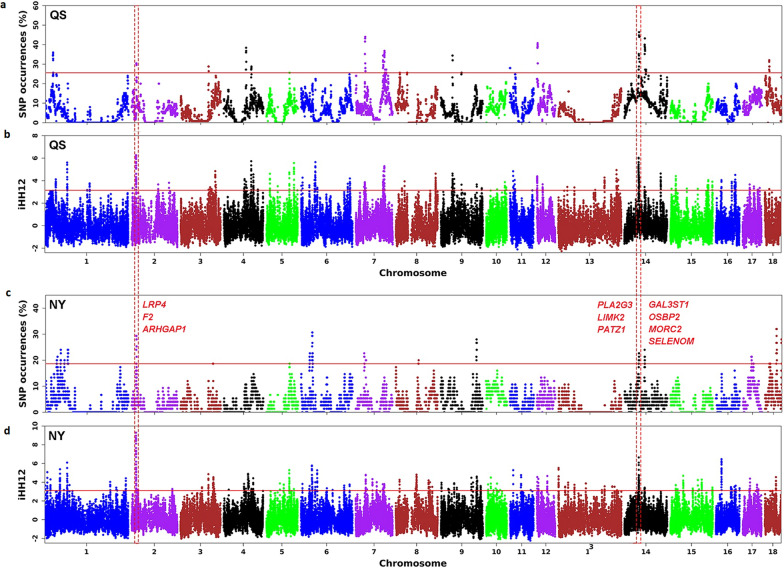
Manhattan plots of the ROH and iHH12 analyses for the Queshan (QS) and Nanyang (NY) breeds. **a** ROH statistics in Queshan; **b** iHH12 statistics in Queshan; **c** ROH statistics in Nanyang; and **d** iHH12 statistics in Nanyang breeds

Two strong selective sweeps on SSC2 (15.49–16.08 Mb) and SSC14 (47.16–48.25 Mb), covering 33 genes, were shared between the Queshan (Fig. 4a and b) and Nanyang (Fig. 4c and d) breeds. Enrichment analysis of these genes yielded seven GO terms (see Additional file 6: Fig. S3C). Regulation of lipid kinase activity was the most significant term, containing the genes *AMBRA1* and *FII* (see Table 2 for full gene names) on SSC2 and *PIK3IP1* on SSC14. Spermatogenesis (GO: 0007283) was another relevant term since it involved a cluster of five functional genes in a 636.35-kb interval on SSC14, including the *LIMK2*, *GAL3ST1*, *PATZ1*, *OSBP2*, and *PLA2G3* genes, which are known to have a role in fertility. It has been shown that knockout or disruption of these five genes contributes to spermatogenic abnormalities in the mouse and to infertility in humans [32–36]. Another gene in this interval, *MORC2* (*MORC2B*), has also been shown to lead to sterility in both sexes in mice [37]. In addition, the *SELENOM* gene within this interval is known to increase weight gain, increase white adipose tissue deposition, and reduce hypothalamic leptin sensitivity in mice [38]. In a small 238.05 kb region on SSC2 under selection, three genes *ARHGAP1*, *FII* and *LRP4* are known to regulate reproduction and growth [39–41]. Information on these 12 genes is provided in Additional file 7: Table S4.

**Table 2:** Gene symbols and full names cited within the text

Gene symbol	Gene full name
*AMBRA1*	*Autophagy and beclin 1 regulator 1*
*FII*	*Coagulation factor II*
*PIK3IP1*	*Phosphoinositide-3-kinase interacting protein*
*LIMK2*	*LIM domain kinase 2*
*GAL3ST1*	*Galactose-3-O-sulfotransferase 1*
*PATZ1*	*POZ (BTB) and AT hook containing zinc finger 1*
*OSBP2*	*Oxysterol-binding protein 2*
*PLA2G3*	*Phospholipase A2, group III*
*MORC2*	*MORC family CW-type zinc finger 2*
*SELENOM*	*Selenoprotein M*
*ARHGAP*	*Rho GTPase activating protein 1*
*LRP4*	*Low density lipoprotein receptor-related protein 4*
*RPTOR*	*Regulatory associated protein of MTOR, complex 1*
*SLC38A10*	*Solute carrier family 38, member 10*
*TEPSIN*	*Adaptor related protein complex 4 accessory*
*CCDC40*	*Coiled-coil domain containing 40*
*CBX2*	*Chromobox 2*
*ADGRB3*	*Adhesion G protein-coupled receptor B3*

### Introgression signals that affect growth performance in Henan indigenous pigs

**Fig. 5:**
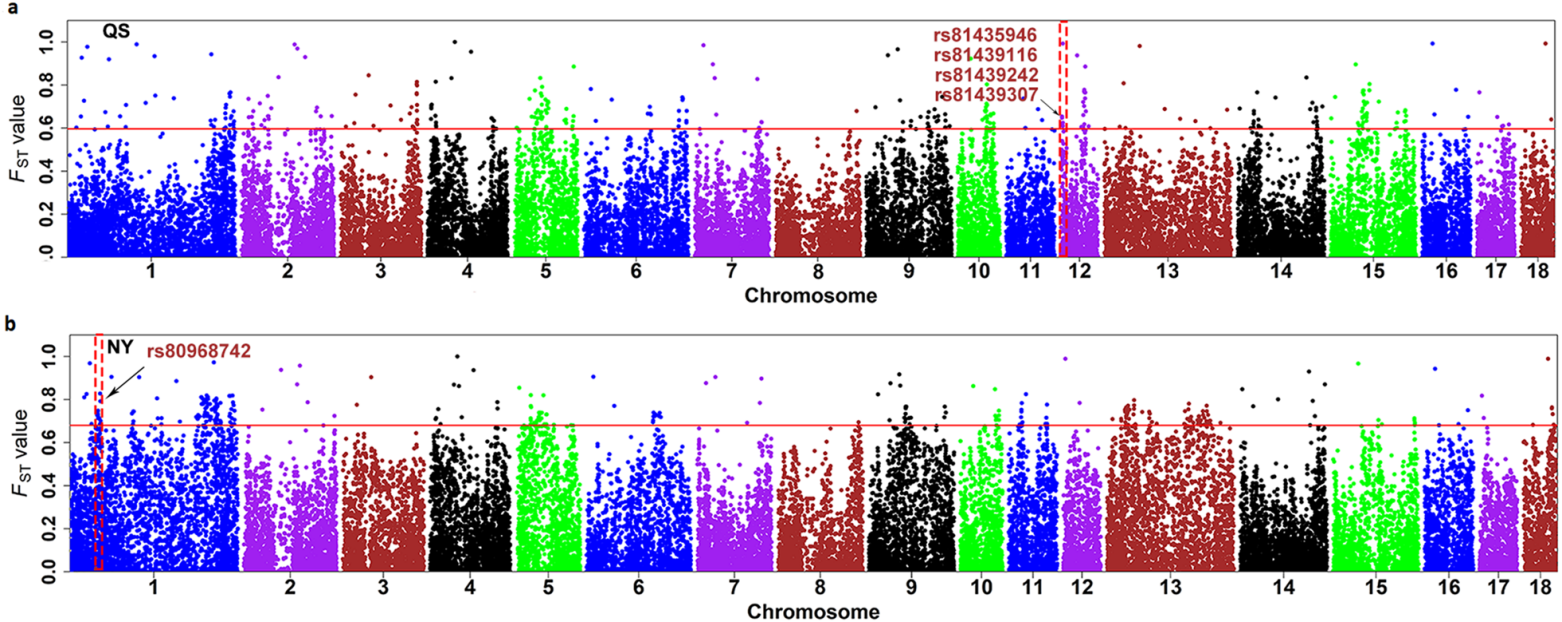
Introgression signals detected in the Queshan (QS) and Nanyang (NY) breeds. **a** Manhattan plots of *F*_ST_ statistics between Queshan and eight Chinese local pig breeds; and **b** Manhattan plots of *F*_ST_ statistics between Nanyang and eight Chinese local pig breeds

**Fig. 6:**
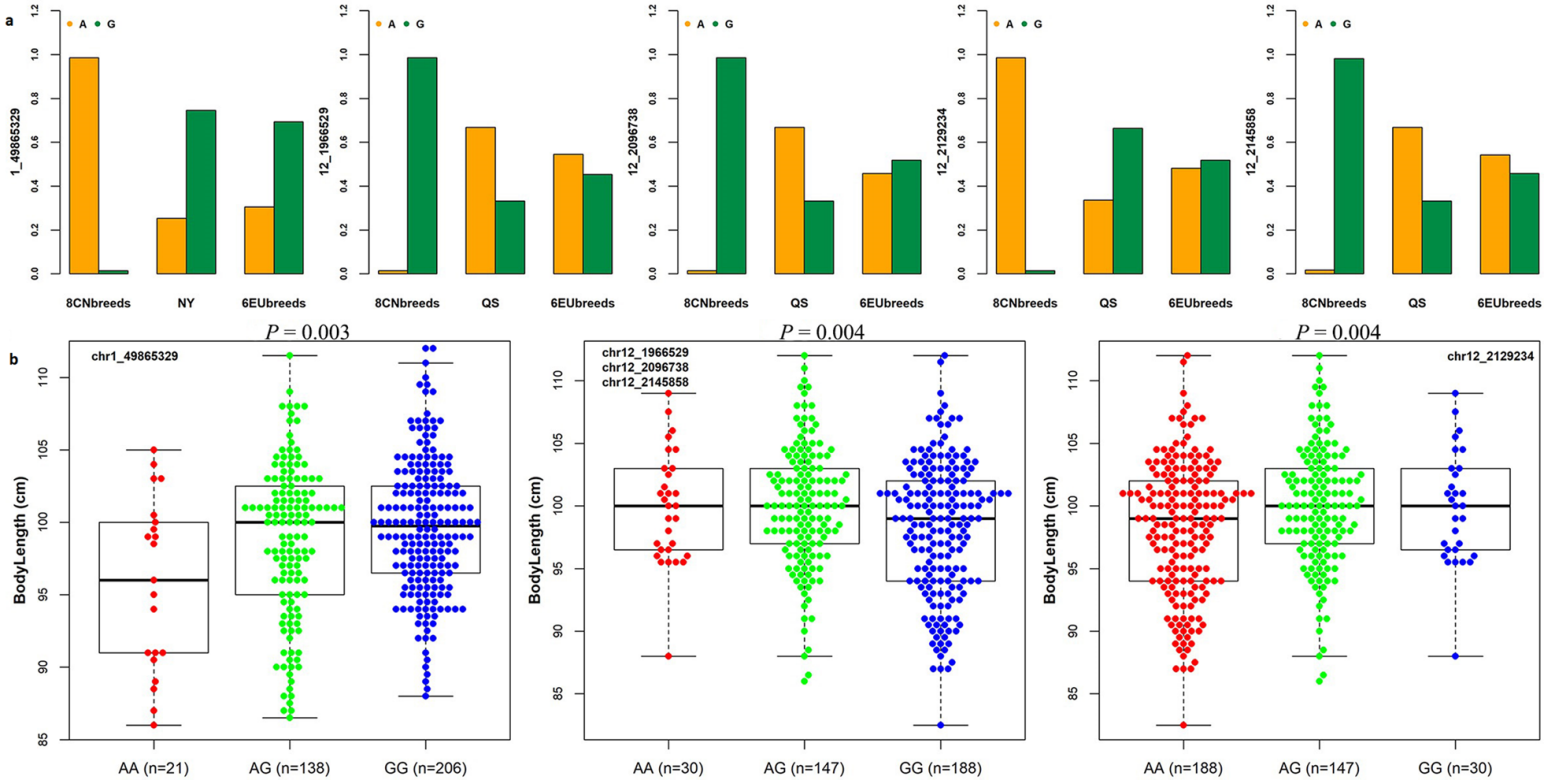
The effects of five significant introgression signals detected in the Queshan (QS) and Nanyang (NY) breeds. **a** Allele frequencies of the five loci in Queshan, Nanyang, eight Chinese local pig breeds and six European pig breeds; and **b** the effects of the five loci on body length in Sujiang pigs

After detecting regions under natural or artificial selection within the Henan native pigs, we used population differentiation detection (*F*_ST_) to investigate whether the genome of the Queshan and Nanyang breeds contained any unique region that differentiated them from the other Chinese pig breeds. In total, 139 individuals from eight representative pig breeds (Meishan, Erhualian, Shaziling, Ganxi, Neijiang, Litang Tibetan, Luchuan and Wuzhishan) were used for this purpose. The common top 1% significant SNPs from the *F*_ST_, ROH, and iHH12 analyses were extracted and are presented in Additional file 5: Fig. S2B and S2C. Four of these SNPs on SSC12 (1.97–2.15 Mb) (rs81435946, rs81439116, rs81439242, and rs81439307) were detected in the genome of the Queshan pigs (Fig. 5a), while one SNP (rs80968742) on SSC1 (49.87 Mb) was detected in the genome of the Nanyang pigs (Fig. 5b). These five loci were almost homozygous for the same allele in eight Chinese pig breeds, while the other alleles were all at high frequency in the Queshan and Nanyang breeds and in the six foreign pig breeds (Fig. 6a).

To investigate the effects of these five SNPs, they were used to genotype 365 nucleus Sujiang pigs for which body size records were available. The Sujiang breed is a hybrid between the Chinese Jiangquhai pig breed and Duroc [42]. The five SNPs were significantly correlated with body length (*P* = 0.004). The high-frequency alleles in the Henan and foreign breeds were the favorable alleles (Fig. 6b). Part of the *RPTOR* gene (SSC12:1,709,126–1,991,560 bp), which lies within the genomic region that is covered by these four SNPs on SSC12 (1.97–2.15 Mb) is known to be associated with body mass index in humans [43], while SNP rs80968742 on SSC1 did not fall within any gene coding region.

After extending the candidate interval under selection for each of the five SNPs by 500 kb upstream and downstream, given the low marker density and the rate of LD decay, and we detected four genes in the extended region on SSC12 (from 1.47 to 2.65 Mb) that are involved in growth and food intake, i.e. *SLC38A10*, *TEPSIN*, *CCDC40* and *CBX2* [44–46], and one gene in the extended interval on SSC1 (from 49.37 to 50.37 Mb), i.e. *ADGRB3*, which has been suggested to cause short stature in humans (https://www.ncbi.nlm.nih.gov/clinvar).

## Discussion

Henan is one of the earliest and the largest pig breeding area in China. In this study, a comparative population genetics analysis was carried out using SNP genotype data from 46 Eurasia-American pig breeds. The obvious separation between the Chinese native pig breeds and foreign pig breeds and a higher consistency of intra-individual branch length in foreign commercial pig breeds support different origins for domestic pigs in Europe and Asia [13, 47, 48] and suggest a smaller intra-individual difference in foreign pig breeds, which have been systematically bred for over a hundred years.

### Classification of local pig breeds in China

In 1986, local pig breeds in China were divided into six types i.e. from North China, South China, East China, Center China, Southwest China, and Plateau China [3]. However, based on the results of the neighbor joining tree, split network, PCA, and admixture analyses, we found that the breeds from Plateau China were mixed with those from Southwest China, i.e. they could not be separated into distinct clusters, like the other four types. The breeds from North China had no separate ancestor of their own but contained lineages from East China, Center China, Southwest China, Plateau China, and foreign pigs. Based on the admixture analysis, we also found that the lineages of the Chinese local pig breeds detected in foreign breeds were mainly of South China origin, as previously reported [47, 48]. Compared to the other foreign pig breeds, the Large White and East China breeds had more genes in common, which all have good reproductive performance.

The geographical distributions of the breeds from Southwest China and Plateau China are very far apart, with those from Southwest China mainly distributed in the Sichuan Basin and the Yunnan-Guizhou Plateau, and those from Plateau China mainly distributed in the Tibet Plateau. The geographical environment and climatic conditions in these areas are complex. The breeds from Southwest China and Plateau China show obvious differences in body size: those from Southwest China are generally larger, with a large head and a wide and concave back and waist, whereas those from Plateau China are more like wild boars, with a shorter body. To date, we cannot explain why, the breeds from Southwest China, i.e. from low altitude regions, were located in the neighbor joining tree among the breeds from Plateau China, i.e. from high altitudes. In addition, genetic adaptation to high altitudes in Tibetan wild boars has been detected through genome comparison of domestic pigs, including Neijiang, with high-altitude wild pigs, including Diqing Tibetan and Gansu Tibetan (see Additional file 3: Table S2) [49].

Among the six types of Chinese native pig breeds, the cluster of breeds from North China is the most dispersed. Their genetic composition is more complex than that of the other breeds, as this cluster not only failed to show a major common ancestor, but it was also mixed with more foreign pig bloodlines, especially the Nanyang and Queshan breeds from the Henan province. This may be due to the northern region of China being the first to raise foreign pig breeds. After 1840, Russian white pigs, Berkshire, and Yorkshire pigs were brought to China by foreigners and they began to be raised in the Northeast and Qingdao, Shandong Province of China [50]. By 1914, the number of hybrid pigs raised in the six northern provinces of China had reached more than 40,000 [51]. Soon after the founding of the People's Republic of China, Henan became the state-owned breeding base of imported commercial pig breeds. Thus, local pigs in the Henan province were more likely to be crossed with foreign pigs to improve production performance. Henan also has a clear record of introduction of Berkshire, Duroc, Landrace, Yorkshire pigs, as well as Neijiang and Ningxiang pigs from Center China, which was confirmed by the results of the admixture analysis. Migration events from Berkshire and Duroc to the Henan breeds were also detected. The Bamei breed, which originates from hybridization of various Huang-Huai-Hai black pigs (including Huainan, Queshan, Laiwu, Hetaodaere, etc.), which form a subgroup of North Chinese breeds with some local pigs at an altitude of 2000 m in the northwest of China, is now closer to its neighbor Gansu Tibetan after 2000 years of natural and artificial selection.

### Genomic signatures of admixture in Henan pig breeds

The breeds from North China, especially the Henan local Nanyang and Queshan breeds have a relatively high genomic heterozygosity, not only because of their ancestral diversity but also because of recent introgression of foreign genetics. Thus, in the analyses of the genetic diversity of current local pig breeds in China, it is necessary to first exclude the influence of genome exchange between Chinese and foreign pig breeds. Considering the large genetic differences within the Nanyang pig population, constant attention must be paid to the dynamics of this population.

The neighbor joining tree shows that the Queshan pigs are divided into two obvious subgroups. There is no evidence that this is related to the difference in head types between the two subgroups (long, short or medium length mouth). Such differences in head type also exist in the East Chinese pigs [52, 53]. Within the Henan local pig breeds, there is relatively little intermixing between the Huainan pigs and external lineages. The Huainan and Erhualian breeds contain a higher level of similar ancestry, maybe because they are geographically close and because, based on the Huainan breeding records, the offspring of the crosses between the Huainan and Erhualian, Huainan and Sutai breeds, respectively, had been used as dams for the Huainan breed to improve reproduction performance and meat quality.

Previous studies have reported that pigs in southern and northern China have different domestication centers. Analysis of the characteristics of the bones of domestic pigs from the Jiahu site in Henan province and the Kuahuqiao site in Zhejiang province also suggested that domestic pigs in northern and southern China originated from different wild ancestors [1]. However, our results show that only the breeds from South China are located at the root of the evolutionary tree of the Chinese local pigs, as was previously reported [12, 47]. In this study, we used SNP genotypes of Chinese native pig breeds from former published studies that use a SNP-array that was designed based mainly on European breeds and which, thus, might be less informative for Chinese pigs and some leave some information undetected. Our results do not provide strong genomic evidence of the ancient role of the northern Chinese pigs. However, we did find that the Henan breeds have an extremely complicated genetic background and share part of their genome with some northern Chinese pig breeds (Erhualian, Hetaodaer, Laiwu, etc.). The breeds from North China are distributed in the vast northern area of China, where two of the three major plains of China are located, the Northeast Plain and the North China Plain. Henan province is located in one of these, with many river resources, is one of the birthplaces of Chinese civilization, and has always been a battleground for military forces. We are not sure whether the rapid dilution of the ancient lineage of the breeds from North China was the result of pig movement associated with migration of people or of the frequent exchange between the modern North Chinese pigs and foreign pigs. Whether this ancient lineage still exists in the Henan local pigs needs to be further analyzed using a higher density SNP array, wider local pig populations, and DNA from wild boar or ancient pigs.

### Genomic signatures of selection of Henan pig breeds

Nevertheless, we detected two interesting genomic regions under selection on SSC14 (47.16 to 48.25 Mb) and SSC2 (15.49 to 16.08 Mb) in both the Queshan and Nanyang breeds. Lipid kinase activity was the top-1 significant enriched term, involving the *FII, AMBRA1,* and *PIK3IP1* genes. To date, there is no evidence that these two genomic regions are related to the immune performance of Henan local pigs. Also worth further in-depth study are five genes involved in spermatogenesis (*LIMK2*, *GAL3ST1*, *PATZ1*, *OSBP2,* and *PLA2G3*), one gene involved in sterility (*MORC2*), and one gene related to fat deposition (*SELENOM*) that were identified within a small region of 636.35 kb on SSC14, and three genes involved in reproduction and growth (*ARHGAP1*, *FII* and *LRP4*) that were identified in a 238.05-kb region on SSC2. We checked these regions on the PigQTLdb (https://www.animalgenome.org/cgi-bin/QTLdb/SS/index) and found some quantitative trait loci and genome-wide association signals related to fat deposition, meat quality, growth, reproduction, and boar taint related hormone levels, etc. In addition, four introgressed SNPs on SSC12 in the Queshan breed and one introgressed SNP on SSC1 in the Nanyang breed that originated from foreign pigs could be associated with growth traits. These chromosomal regions contain growth-related genes such as *RPTOR* and *ADGRB3* and thus should be further investigated. All Henan local pigs are fatty ancient local pig breeds with coarse feeding tolerance and strong fat deposition capacity. Thus, the selection signals associated with lipid kinase activity, nutrition level, and immune-related terms in the Queshan and Nanyang breeds are also worthy of further study. It would be interesting to perform genetic association studies in these regions for traits such as lipid kinase activity, sperm quality, fat deposition, growth traits, and immune-related traits in Henan native pig breeds to detect candidate SNPs for marker-assisted selection.

### Two main subgroups of East China pigs

Moreover, our results show that the breeds from East China are divided into two subgroups, i.e. the all-black pigs (with the exception of the Meishan breed, which has white trotters), and the spotted and two-end black pigs, which is basically consistent with the geographical distribution of these breeds. The breeds from East China are distributed in the Han River and in the middle and lower reaches of the Yangtze River regions at the junction between the North China and Center China areas [3]. The breeds from East China, also known as the North China and Center China transitional pig breeds, are basically bred by crossing breeds from North China and Center China [54, 55]. This separation is consistent with a statement that under the influence of breeds from both North China and Center China, the breeds from East China, which include many varieties can be divided into two categories. One is greatly influenced by the neighboring Northern Chinese pigs with large sagging ears, a sunken back and waist, robust limbs, and thick wrinkled skin, while the other is greatly influenced by the Center Chinese pigs that show a wide coat color variation, from whole black to spotted [56].

## Conclusions

Our results show that the Henan native pigs have a complex genetic background and share some common lineage with representative pig breeds from northern and eastern China, although this evidence is not sufficient to support Henan as an early center of pig domestication in China. Second, we found that, compared with other local pig breeds in China, Henan local pig breeds contained more foreign pig lineage and had a higher level of heterozygosity and greater genetic diversity. Third, we detected two interesting selective sweeps associated with lipid kinase activity, reproduction, growth, and fat deposition, and identified five introgressed SNPs from foreign pigs that are associated with growth in Henan pigs. Finally, our results show that the breed types from Southwest China and Plateau China can not be distinguished; that, after 2000 years of natural and artificial selection, the Bamei breed is genetically far from its North China pig origin; and that the breeds from East China can be divided into two subgroups based on their geographic distribution and genetics.

### Supplementary Information

**Additional file 1: Figure S1.** Distribution of six types of Chinese local pig breeds and 40 Chinese pig breeds used in this study.

**Additional file 2: Table S1.** Records of the pig breeds introduced in the Nanyang, Zhumadian and Xinyang areas of the Henan province.

**Additional file 3: Table S2.** Genetic diversity of 46 Eurasian pig breeds used in this study [3].

**Additional file 4: Table S3.** F3 analysis results of Henan native pig breeds.

**Additional file 5: Figure S2.** Venn diagram of signatures of selection detected by ROH and iHH12 in Queshan (QS) and Nanyang (NY) pigs. A, Signatures of selection in Queshan and Nanyang pigs. B, Signatures of selection in Queshan pigs. C, Signatures of selection in Nanyang pigs.

**Additional file 6: Figure S3.** GO terms enrichment heatmap of signatures of selection detected by the ROH and iHH12 methods in Queshan and Nanyang pigs. A, Queshan pigs. B, Nanyang pigs. C, Queshan and Nanyang pigs.

**Additional file 7: Table S4.** Candidate genes under selection in both Queshan and Nanyang pigs.

## Data Availability

All datasets used in this study are available from the corresponding author on reasonable request.
