# Supplementary figures and images for: Potential selection for lipid kinase activity and spermatogenesis in Henan native pig breeds and growth shaping by introgression of European genes
Source: Genet Sel Evol. 2023 Sep 18;55:64. doi: 10.1186/s12711-023-00841-y (PMC10506266; doi:10.1186/s12711-023-00841-y)

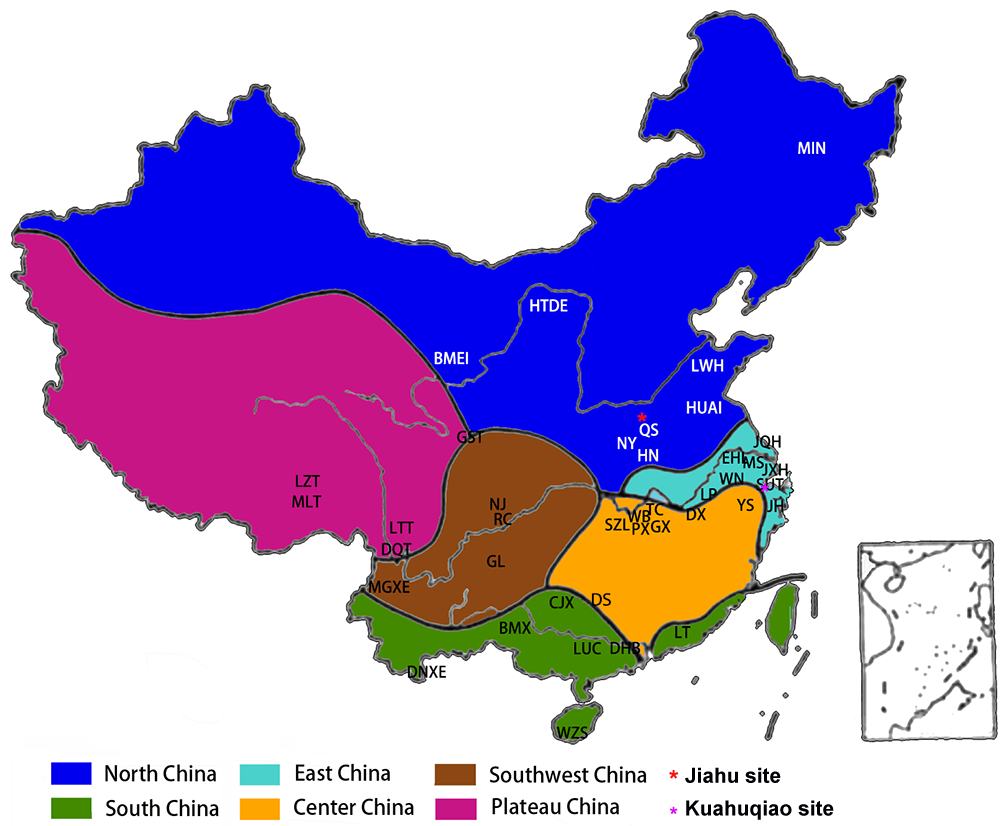

Supplement: Supplementary file 1 — Additional file 1: Figure S1. Distribution of six types of Chinese local pig breeds and 40 Chinese pig breeds used in this study. [file 12711_2023_841_MOESM1_ESM.tif]

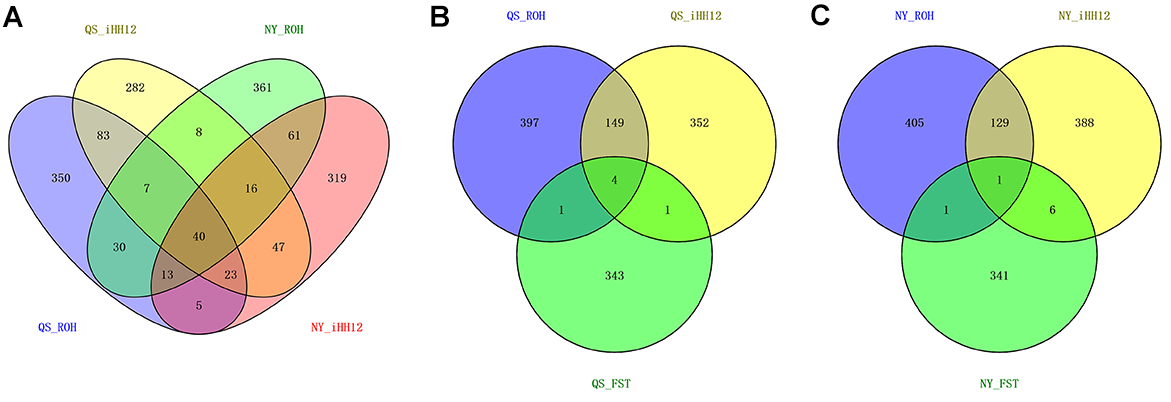

Supplement: Supplementary file 5 — Additional file 5: Figure S2. Venn diagram of signatures of selection detected by ROH and iHH12 in Queshan (QS) and Nanyang (NY) pigs. A, Signatures of selection in Queshan and Nanyang pigs. B, Signatures of selection in Queshan pigs. C, Signatures of selection in Nanyang pigs. [file 12711_2023_841_MOESM5_ESM.tif]

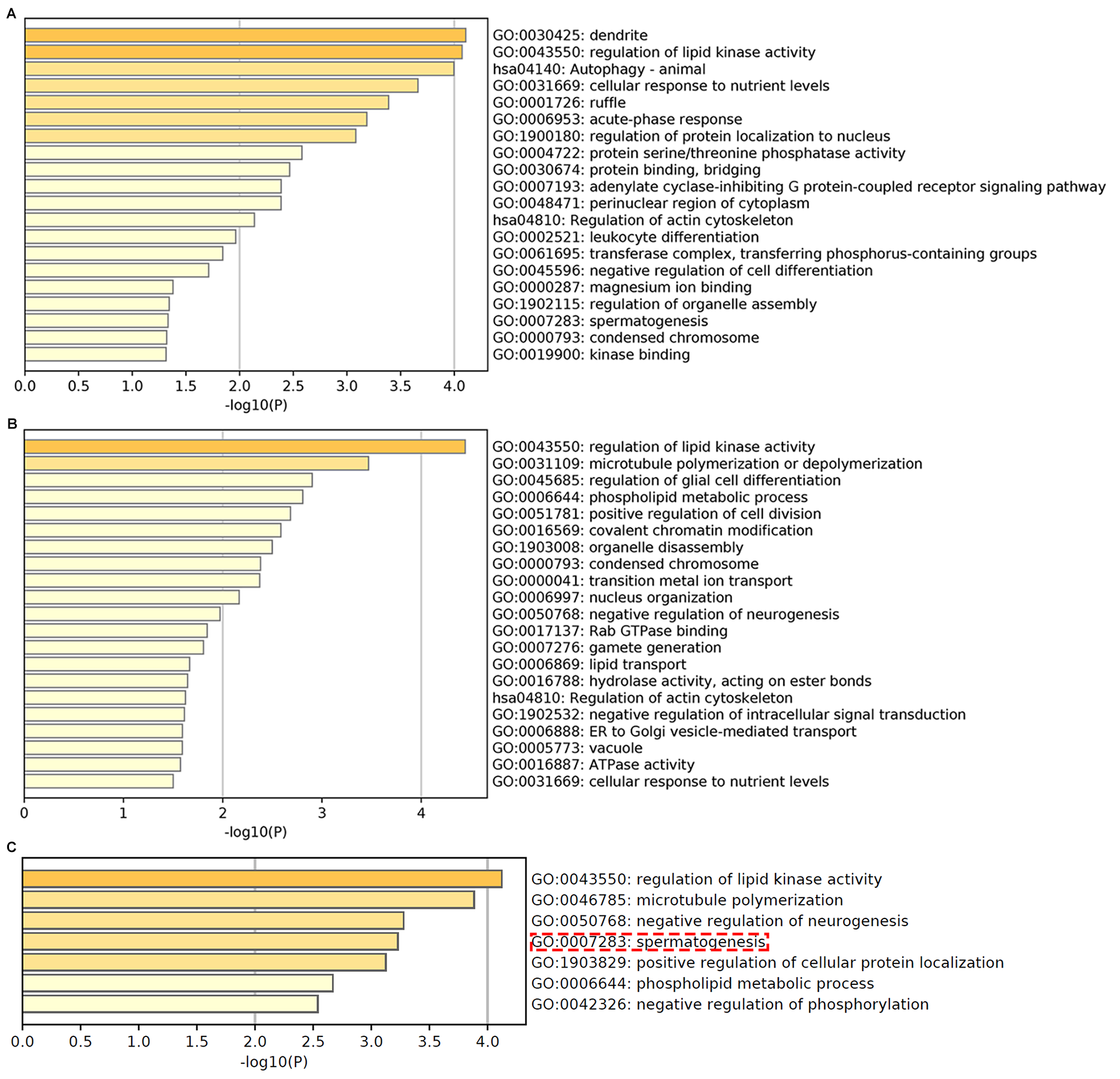

Supplement: Supplementary file 6 — Additional file 6: Figure S3. GO terms enrichment heatmap of signatures of selection detected by the ROH and iHH12 methods in Queshan and Nanyang pigs. A, Queshan pigs. B, Nanyang pigs. C, Queshan and Nanyang pigs. [file 12711_2023_841_MOESM6_ESM.tif]
